# Sibutramine-Induced Nonischemic Cardiomyopathy

**DOI:** 10.7759/cureus.21650

**Published:** 2022-01-26

**Authors:** Meet S Shah, Zeel K Patel, Ronak Bharucha, Tirth Talati, Michael Benz

**Affiliations:** 1 Internal Medicine, Rutgers University New Jersey Medical School, Newark, USA; 2 Internal Medicine, University at Buffalo, Buffalo, USA; 3 Internal Medicine, Hackensack University Medical Center, Hackensack, USA; 4 Interventional Cardiology, Christ Hospital, Jersey City, USA

**Keywords:** nonischemic, anti-obesity, dietary supplement, cardiomyopathy, sibutramine

## Abstract

Within the past 20 years, the global pandemic of obesity and associated life-threatening comorbidities significantly promoted the development and intervention of anti-obesity pharmacotherapy. Sibutramine hydrochloride monohydrate, formerly sold under the brand name Meridia and Reductil among others, is an anti-obesity, selective serotonin, and norepinephrine reuptake inhibitor drug that suppresses appetite and reduces body weight in conjunction with lifestyle modifications. However, since 2010, it has been discontinued in a majority of countries such as the United States and European Union due to an associated increase in cardiovascular events such as hypertension, tachycardia, arrhythmias, and myocardial infarction. Thus, this article illustrates a case of sibutramine-induced nonischemic cardiomyopathy, including details of evaluation, management, and monitoring of patient progress. Herein, we present a case report of a 19-year-old male with no prior medical conditions who presented to the emergency department after being found in a state of cardiac arrest (pulseless ventricular fibrillation) with consequent intubation in the field. Upon admission, cardiac catheterization and echocardiography revealed patent coronary arteries with a reduced ejection fraction of approximately 15%-20%. Acute systolic heart failure secondary to nonischemic cardiomyopathy was treated with standard medical management. In addition, due to continued episodes of non-sustained ventricular tachycardia, the patient also underwent a subcutaneous implantable cardioverter-defibrillator (ICD) placement.

## Introduction

Obesity is a chronic disease that can be defined as an excessive accumulation of body fat [1]. Since 2016, the World Health Organization (WHO) has reported an increasing prevalence worldwide, affecting more than 650 million people. The lack of effective weight loss treatments, based solely on lifestyle modifications such as diet and exercise, has promoted the development of anti-obesity pharmacotherapy as a supplemental treatment. Sibutramine hydrochloride monohydrate is an anti-obesity drug that suppresses appetite and reduces body weight in conjunction with lifestyle modifications. By selectively inhibiting the presynaptic uptake of monoaminergic neurotransmitters serotonin and norepinephrine, increasing neuropeptide release in the arcuate nucleus, and preventing the decrease of basal energy expenditure following weight loss, sibutramine proved to reduce weight in those patients who were obese and/or overweight [2]. However, due to an increase in cardiovascular events and strokes, sibutramine was discontinued in the majority of countries, such as Australia, the United States, and the European Union, in 2010. The Sibutramine Cardiovascular Outcomes Trial (SCOUT) confirmed that subjects with preexisting cardiovascular disease on long-term (five years) treatment with sibutramine (10-15 mg/day) had a significantly increased risk for nonfatal myocardial infarction and stroke, but not cardiovascular death or all-cause mortality [3]. Since 2002, several cardiovascular adverse events, such as hypertension, tachycardia, cardiomyopathy, arrhythmias, and myocardial infarction, have been reported [4]. In particular, one adverse effect caused by sibutramine includes nonischemic cardiomyopathy. As a myocardial disorder in which the heart muscle is structurally and functionally abnormal in the absence of coronary artery disease (CAD), hypertension, valvular disease, and congenital heart disease, nonischemic cardiomyopathy can be caused by several factors, including viral infection, drug reactions, infiltrative processes, or autoimmune diseases. Thus, in this article, we report a unique case of sibutramine-induced nonischemic cardiomyopathy with details of symptoms, clinical findings of diagnostic testing, and medical management.

## Case presentation

A 19-year-old male patient with a past medical history of obesity and no family history was found in a state of ventricular fibrillation and was subsequently brought to the hospital by emergency medical services. At that time, he was engaged in his daily activities, sitting comfortably at a barbershop when he felt dizzy and had a syncopal episode. Upon arrival to the scene, EMS personnel defibrillated him, which immediately converted his rhythm into pulseless ventricular tachycardia. Another defibrillation was administered, which converted the patient’s rhythm into ventricular tachycardia with pulse. EMS personnel then gave him a dose of amiodarone en route to the hospital. Echocardiogram (ECG) in the field illustrated ST elevations in inferior and lateral leads (leads II, V4-V6). At the hospital, while the patient was transferred from the emergency room to the cardiac catheterization laboratory, he was given aspirin 325 mg and ticagrelor 180 mg. He was then started on an IV amiodarone drip with bolus and a normal saline bolus. Pertinent social history includes the use of hookah and E-cigarettes, but no alcohol or illicit drug use. According to the patient’s family, the patient started a diet 12 months prior in order to lose weight and successfully lost 100 pounds. He also used diet pills, namely, sibutramine hydrochloride monohydrate, to enhance his weight loss process. After six months of use, he had discontinued using the drug without any significant reasoning and had not used it again for the past six months.

**Figure 1:**
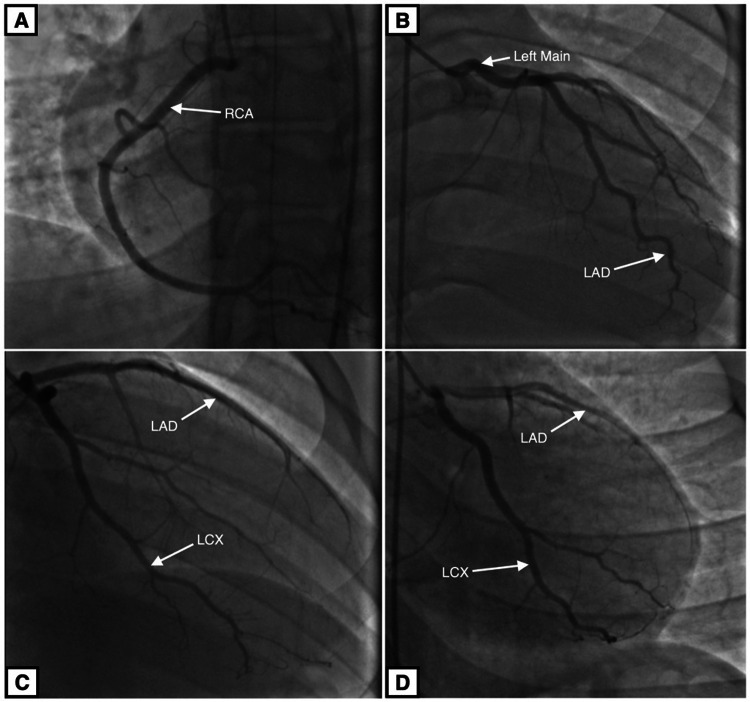
Coronary angiogram Coronary arteries are patent, without any morphological abnormalities. A: Right coronary artery (RCA). B: Left main coronary artery and left anterior descending (LAD) artery. C: Left anterior descending artery and left circumflex (LCX) artery. D: Left anterior descending artery and left circumflex artery.

**Figure 2:**
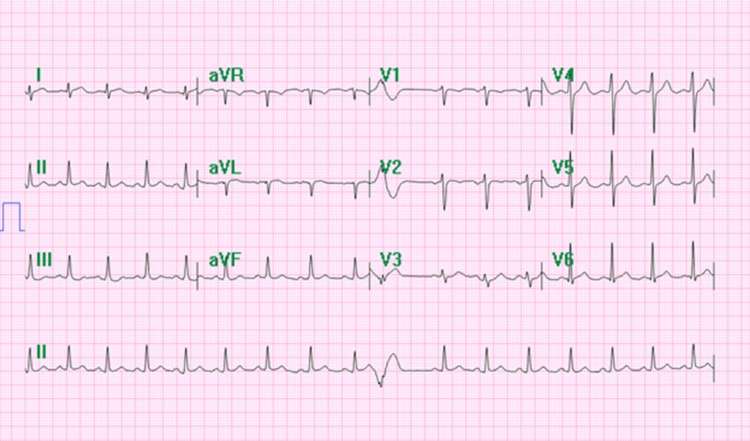
Electrocardiogram ECG tracing in the hospital shows a normal rate, sinus rhythm, and premature ventricular contraction.

The patient underwent a left heart catheterization and coronary angiogram (Figure 1), which showed the following results: left main coronary, patent; left anterior descending (LAD) and diagonal branches, patent; left circumflex and obtuse marginal branches, patent; right coronary artery, patent; and dominant, dilated left ventricle and severe global hypokinesis with an estimated ejection fraction of 15%. Hospital ECG was unremarkable with the exception of premature ventricular complex (Figure 2). The patient was found to have severe left ventricular systolic dysfunction and required vasopressors to maintain adequate blood pressure. He was put on a mechanical ventilator and transferred to the intensive care unit, with acute respiratory failure secondary to his cardiac arrest.

In the intensive care unit, the patient had episodes of non-sustained ventricular tachycardia. Amiodarone and lidocaine treatment failed to control his arrhythmia. In addition, ventricular tachycardia episodes became more frequent. Due to the failure of medical management, the patient was transferred out to a different hospital for electrophysiology studies. An electrophysiology study (EPS)/ablation study showed normal functioning of the sinus node, atrial chamber, AV node, His-Purkinje system, and ventricular chamber. Consequently, he underwent a subcutaneous implantable cardioverter-defibrillator (ICD) placement. The remainder of the hospital course was unremarkable for any significant cardiovascular events, and the patient was discharged to a subacute rehabilitation center on discharge.

## Discussion

Cardiomyopathy refers to a subset of diseases affecting the myocardium, which manifests clinically with heart failure symptoms. It can be broadly classified into two categories: ischemic and nonischemic cardiomyopathy. Ischemic cardiomyopathy is caused by ventricular dysfunction due to myocardial ischemia and infarction due to stenosed coronary arteries. Nonischemic cardiomyopathy encompasses broader causes, without any specific underlying pathophysiology, and has a lengthy differential diagnosis. The American Heart Association defines nonischemic cardiomyopathy as a disease of the myocardium associated with mechanical or electrical dysfunction, exhibiting inappropriate ventricular hypertrophy or dilation. Some of the potential contributing factors are hemodynamic pathology, infection, toxic injury, and immunologic dysfunction. Up to 40% of cardiomyopathy can be nonischemic in etiology, as studied in a population of 156,000 hospitalized patients with heart failure across 319 US hospitals from 2005 to 2013 [5]. A growing number of nonischemic disorders are being recognized as genetic causes and classified as either primary or secondary due to either intracardiac or systemic disease, respectively.

Here, we present an atypical case of a 19-year-old individual with a past medical history of obesity and no family history who presented with a syncopal episode followed by cardiac arrest (pulseless ventricular fibrillation) and a field ECG that showed ST elevations in the lateral leads. The patient was defibrillated, and standard medical management was resumed as the patient was brought to the cardiac catheterization laboratory for left heart catheterization and coronary angiogram. Upon further diagnostic workup, he was found to have severely reduced ejection fraction for his age with normal coronaries. As a result, he was ultimately diagnosed with nonischemic cardiomyopathy with a background of sibutramine treatment as the only possible risk factor for his condition. He was able to successfully obtain this dietary supplement from the Dominican Republic.

Sibutramine is an appetite suppressor that works by inhibiting the reuptake of monoamines, specifically norepinephrine, serotonin, and dopamine, producing an overall increase in satiety. It has two active metabolites: desmethylsibutramine and didesmethylsibutramine. This drug was initially approved by the FDA in 1997 for the treatment of obesity, but there has been a growing list of adverse effects in the years since. The most common side effects include dry mouth, nausea, constipation, hypertension, trouble sleeping, and joint pain. Infrequent but more serious side effects include heart attacks, strokes, cardiac arrhythmias, chest pain, paresthesias, seizures, gastrointestinal bleeding, and jaundice. The SCOUT study examined sibutramine use in conjunction with a weight management program in patients with high risk for heart disease [3]. It showed those patients to be at an increased risk of cardiovascular complications such as nonfatal myocardial infarction leading to cardiomyopathy and nonfatal stroke, but not an overall increased risk of cardiovascular death or all-cause mortality. However, the study did not consider patients who are obese but free of cardiac diseases, such as our patient presented above. It has also been shown that due to its specific mechanism of action as a sympathomimetic agent, sibutramine tends to cause a moderate increase in heart rate that may attenuate a reduction in blood pressure due to weight loss or even lead to overall increased blood pressure [6]. Harrison-Woolrych et al. (2006) investigated multiple case reports and World Health Organization (WHO) database for post-marketing surveillance, which showed an association between sibutramine treatment and QT interval prolongation leading to fatal cardiac arrhythmias such as torsade de pointes, ventricular tachycardia, and ventricular fibrillation [7]. In 2010, the FDA stated that sibutramine should not be used in patients with a history of cardiovascular diseases, including CAD, a stroke or TIA, heart arrhythmias, CHF, or uncontrolled hypertension.

## Conclusions

The 19-year-old patient with a past medical history of obesity and no significant cardiac family history who presented with a syncopal episode followed by cardiac arrest was eventually diagnosed with nonischemic cardiomyopathy. A detailed history from the patient and his family, as well as other diagnostic workups during the hospitalization, ruled out other major common causes of nonischemic cardiomyopathy, suggesting drug-induced cardiomyopathy as the most likely cause of this patient’s presentation.

Ultimately, his cardiomyopathy resulted in the patient receiving an ICD placement due to failure of medical management to properly restore his cardiac function. Since his hospital course, the patient has been recovering well and performing his routine activities on a daily basis. Although the patient discontinued sibutramine approximately six months prior to this illness, this case of nonischemic cardiomyopathy attributed to the likely side effect of the drug demonstrates the presence of cardiovascular disease risk even after discontinuation of the drug. Thus, the risk of nonischemic cardiomyopathy and cardiac arrhythmias is not completely alleviated with the discontinuation of this drug.
